# A conceptual framework for culturally appropriate advocacy with racialised groups

**DOI:** 10.3389/fpsyt.2023.1173591

**Published:** 2023-07-11

**Authors:** Anthony Salla, Karen Newbigging, Doreen Joseph, Emachi Eneje

**Affiliations:** ^1^City University of London, London, United Kingdom; ^2^Department of Psychiatry, University of Oxford, Oxford, United Kingdom; ^3^School of Social Policy, University of Birmingham, Birmingham, United Kingdom

**Keywords:** epistemic injustice, advocacy, culturally appropriate, mental health legislation, race, ethnicity, social justice, independent mental health advocacy

## Abstract

Independent mental health advocacy was introduced in England to protect and promote the rights of people detained under mental health legislation. However, shortcomings in access and delivery to racialised people, raising concerns about equity, were identified by a review of the Mental Health Act. The development of culturally appropriate advocacy was recommended. While the term culturally appropriate may be taken for granted it is poorly defined and limited efforts have conceptualized it in relation to advocacy. Ideally, advocacy operates as a liberatory practice to challenge epistemic injustice, which people experiencing poor mental health are at acute risk of. This is amplified for people from racialised communities through systemic racism. This paper argues that advocacy and culturally appropriate practices are especially relevant to racialised people. It clarifies the importance of culture, race and racism to the role of advocacy, and understanding advocacy through the conceptual lens of epistemic injustice. A central aim of the paper is to draw on and appraise cultural competency models to develop a conceptual framing of cultural appropriate advocacy to promote epistemic justice.

## Introduction

This paper is concerned with independent mental health advocacy (IMHA) required by the Mental Health (Amendment) Act (MHA) 2007. In England after 2007, the purpose of this form of advocacy is to protect and promote the rights of people detained under the 1983 Mental Health Act. Advocacy is especially relevant to racialised groups, who face the double discrimination associated with their mental health status coupled with a racialised identity. Their worse experience and outcomes in relation to mental health services has been well documented and the need for advocacy to address this identified more than 20 years ago (1).

A study of the early implementation of IMHA identified that those people who most needed an advocate, and this included people from racialised communities^1^, were the least likely to access one (2, 3). Furthermore, the Review of the Mental Health Act identified shortcomings in IMHA provision for people from specific racialised communities and recommended the introduction of culturally appropriate advocacy (CAA) (4).

This paper draws on the extant literature, and the experience of the authors in scoping and undertaking an evaluation of pilots to introduce CAA (5) to put forward a conceptual framing for CAA. It argues that the framing offered holds potential to improve conditions of epistemic injustice experienced by racialised people in mental healthcare.

In the background section, epistemic injustice and advocacy are briefly covered to pinpoint their relevance to mental healthcare and their parallel aims in addressing power asymmetries. The following section highlights a gap in the literature in relation to the conceptual framing of CAA. The paper then proceeds in the hope of starting a conversation about CAA. It begins by clarifying some of the core elements of culture and reasserting its importance in relation to mental wellbeing and poor mental health, and the specific relevance of cultural beliefs around rights and epistemic justice to advocacy. It moves on to discuss the necessity for any notion of culturally appropriate to be undergirded by an emphasis on dealing with race and racism. The paper then offers a critique of cultural competency and uses this to inform a definition of cultural appropriate advocacy. Drawing on the literature, steps are taken to propose ‘possible’ learning domains at an individual level, alongside organizational considerations to conceptually frame culturally appropriate advocacy as a mechanism to challenge epistemic injustice.

## Background

### Epistemic injustice

The theory of epistemic injustice developed by Fricker (6) as a foundation for social injustice has been identified as important in understanding and addressing the power asymmetries between survivors/service users and mental health professionals (7). Fricker conceptualized epistemic injustice as the wrong done to a person “*specifically in her capacity as a knower*” (2007: 18) reflecting prejudice based on social identity. Fricker distinguishes two forms of epistemic injustice, which she now refers to as discriminatory epistemic injustice (8) - testimonial and hermeneutic.

Testimonial injustice is a form of prejudice where the speaker is misjudged as a knower and whose credibility is seen as lesser, i.e., ‘direct discrimination (2017, 53) (8) such that their testimony is ignored, dismissed or downgraded as being unreliable. This then justifies exclusion from decision-making about their lives and potentially compulsion (9, 10). Hermeneutic injustice, is a form of indirect discrimination, whereby the interpretation and meaning of experience, is disadvantaged by a lack of conceptual resources to understand the experience (11) or where forms of knowledge do not exist, are not fairly distributed or are marginalized reflecting dominant social norms (12).

As Hill Collins observes, epistemologies are embedded within social institutions and their practices, thus “accomplishing social inequality relies upon strategies of epistemic injustice that collectively reproduce epistemic oppression” (2017: 118) (13) Thus, mental health service user/survivor knowledge is subjugated to the ‘official’, i.e., normative, version that reflects dominant values and beliefs, described by LeBlanc and Kinsella as a form of pre-emptive silencing as a consequence of sanism (14) The power asymmetry in the value accorded to knowledge between mental health professionals and survivors/service users is clear (15), and very evident in the, often egregious, experience of people from racialised communities in relation to statutory mental health services (16). For people from racialised communities experiencing poor mental health, the knowledge, values and beliefs embedded in the mental health system not only reflect “a historical failure to draw on knowledge-systems in non-Western cultures” (2017: 31) (17) but also systemic racism and the hegemonic power of whiteness (18, 19).

### Advocacy as a liberatory practice

People who experience poor mental health face a serious risk of epistemic injustice, with their knowledge and experience being dismissed or discredited, underpinning social inequalities. This risk is amplified by race, gender, sexual identity, and/or disability due to structural inequalities reflected in social processes of marginalization and discrimination. In this context, advocacy is situated within a challenging territory needing to situate cultural differences within the realms of socio-political-structural forces while ensuring service users are heard and their rights upheld.

Advocacy has been described as a liberatory practice because it is concerned with enabling marginalized voices to be heard and associated forms of knowledge possible (20). It takes many different forms including as social movements for greater justice and in mental health has evolved from collective action to an emphasis on individual level provision (21). Nonetheless, advocacy’s roots are founded on the acknowledgement of power disparities, and the need for greater control and choice in relation to public services (22–24). Advocates, therefore, operate at the junction where differing conceptualisations of distress and rights are buttressed against the dominance of psy-disciplines (3, 25, 26).

Advocacy, as ‘a liberatory practice (20), can be viewed as a mechanism to address epistemic injustice by ensuring that the testimony of people and alternative meanings of experience and preferences for support are promoted and heard (12). The view that health professionals are epistemically privileged by virtue of their access to specialized knowledge (27) has been contested through the activism of people with lived experience and the development of mad studies (15). Indeed, this has been countered by the recognition of the epistemic privilege of people who have lived experience of mental distress (28). Advocacy, by giving voice to meaning and experience, has the potential to democratize the relationships in care provision by reducing power asymmetries apparent in healthcare systems and fostering greater inclusion. It can be viewed as a critical component of an equitable approach, shifting power dynamics to ensure greater accountability and an equal basis for service users in decision-making.

Using findings from an evaluation of IMHA services in England, Newbigging and Ridley (12) concluded that advocacy can serve to legitimate the voice of people detained under mental health legislation, and thus, go some way to achieving testimonial justice. However, their analysis suggests that advocacy had little impact on achieving hermeneutic justice. They posit two reasons for this: first the context of compulsion and the associated feelings of fearfulness and disempowerment engender compliance with the dominant narrative of mental distress, and second, the increasing professionalization of advocacy has impacted on forms of advocacy that have stronger connections with activism and survivor/service user led groups. If advocacy is to realize its potential for addressing both forms of epistemic injustice attention needs to be paid to this in conceptualizing and implementing CAA.

### A recommendation for culturally appropriate advocacy

Despite the valuable role advocacy can play in rebalancing power, a body of literature suggests that racialised groups are not content with advocacy provision (29). Research indicates there is limited engagement among racialised groups, and present models of advocacy are incongruent with their interests and concerns; such groups, for example, expressed greater emphasis on rights being asserted through activism and the need for collectivist practices (5). This contrasts with the mainstream model of statutory advocacy which is largely reactive to abuses, and gives primacy to individualism, independence and autonomy (30). While commissioning models and austerity can influence practice, this dissonance, alongside concerns about advocacy provision (31), and longstanding racialised inequality in mental healthcare, have led to calls for CAA (4).

CAA was recommended by the Independent Review of the Mental Health Act, to address the disproportionate rates of detention of racialised people and to improve their experience of mental health services (4). While calls for culturally specific initiatives are justified (32–35), the term itself is nevertheless enigmatic; it lacks robust definition (36) and there is minimal evidence on how it is translated into advocacy provision. Despite four decades of attention, reviews have found significant variation in what practitioners’ feel cultural competence means in practice and to professional standards (37). Therefore, unpacking and comprehending such ambiguity can assist our efforts to conceptualize CAA.

### Centralizing culture, race, and racism to the role of advocacy

#### Embracing culture

Mollah suggests a problem implementing cultural diversity initiatives stems in part from confusion about defining the term culture itself (37). Certainly, being culturally appropriate is not something exclusive to racialised diversity, however there has been particular emphasis on racialised people because of their negative experiences and poor outcomes in relation to public services. As Richardson and Fulton (38) comment ‘[a]lthough cultural competence is an inclusive notion it is especially important in relation to Black and minority ethnic communities because of their particular, rather than exclusive, needs’ (p. 10).

Cultural appropriateness stems from the idea that specific and diverse needs are being met, and a service is grounded in commitment to equitable practices. Yet, culture is a challenging concept to come to grips with, public discourse conflates it with definitions of race and ethnicity, which are in themselves convoluted and imprecise. It is a nebulous and intangible term; Johada (39) described it as an elusive concept that can be whatever a person wants it to be. Culture conjures up various meanings and academic disciplines have their own slant on how it is interpreted (40, 41). Given this degree of uncertainty one may ask: how is culture relevant to racialised groups and advocacy?

Despite ambiguities about its meaning there is agreement that core elements form how we understand and define culture. Castro contends these include: common heritage and history passed from one generation to the next; shared values, beliefs, customs, behaviors, traditions, institutions, arts, folklore and lifestyle; similar relationship and socialization patterns; a common pattern or style of communication or language; geographic location of residence (e.g., country; community; urban, suburban, or rural location); and patterns of dress and diet (42). Of particular relevance is how culture exists in people’s minds, which Holstede (43) proposed is part of our mental software.

For the purposes of this paper, we use a definition of culture that engenders a sense of cohesion between groups through shared patterns of belief, feelings and adaptation which people carry in their minds (44). This is illustrated by Spencer-Oatey (45) who states:

Culture is a fuzzy set of basic assumptions and values, orientations to life, beliefs, policies, procedures and behavioural conventions that are shared by a group of people, and that influence (but do not determine) each member’s behaviour and his/her interpretations of the ‘meaning’ of other people’s behaviour. (p. 3)

This way of understanding culture is aligned with UNESCO’s definition which sees culture as being based on ‘distinctive spiritual, material, intellectual and emotional features of society or social group’ (46). A non-essentialist stance is taken which views culture as being based on subjective perspectives; in doing so it presumes that cultural manifestations are dependent on context as individuals create and negotiate varied circumstances. In this way people from racialised groups may adhere to practices that are constructed as being culturally specific while others may not act in accordance with these codified ways.

Although culture is about similarities between groups and codified similarities, it can never be understood as being static and impenetrable as in an ever increasingly interconnected world cultures exist side by side influencing and informing one another’s ways of living. Nevertheless, a position is taken that culture structures the way people view the world and the sets of beliefs, norms, and values concerning the nature of relationships, the way people live their lives, and the way people organize their environments. While this provides a framework for discussion, and while appreciating that culture is a fuzzy and broad concept, elements described here are pertinent to the diversity advocacy must embrace.

### Culture and mental health

Although culture is a fluid concept it is a central part of how we understand mental health due to its established influence on health practices. As Hernandez remarks, “Culture influences what gets defined as a problem, how the problem is understood and which solutions to the problem are acceptable” (p.1047) (47). It has multi-layered dimensions which interact with class, religion, language, nationality and gender (48), each of which impinge on the way an individual engages with and experiences mental health services.

Mental health professionals and advocates must consider different dimensions of culture in their search for quality care. These dimensions can be physically observable, including forms of address, ceremonies and rituals, food, dress and music. It is nevertheless vital to have a deeper understanding of cultural forms which are hidden. People’s assumptions, non-verbal cues, deeply embedded thoughts, perceptions, unconscious feelings and underlying assumptions are all part of culture (49). Various reviews (50, 51) have looked at the relationship between culture and mental health and highlighted its importance to the delivery of mental healthcare. Culture has been shown to influence emotional expression (52), idioms of distress (53) and assumptions about attitudes and responses to pain (54, 55), including levels of shame, which in turn influences help-seeking and engagement with professionals (56–58).

Culture informs people’s ideas about hierarchical power structures which can have implications for autonomy within therapeutic relationships. Cultural differences are apparent in notions of collectivism as some groups are more likely to have community support structures which can be helpful for coping; while for some groups spirituality can be more pivotal to illness behavior (59–61). Evidently culture plays a central role in mental healthcare; misunderstandings can lead to reduced levels of trust and confidence (62).

Cultural differences can result in misinterpretations of experience and a dismissing of forms of support that people from racialised communities value. This is in a context of ‘white’ models of illness, assessment, care and treatment, and the impact of colonialism (63). Fricker’s concept of epistemic injustice has been used to describe the downgrading or dismissing of experience on the basis of mental health status, reflecting a presumption of irrationality (8), and privileging dominant discourses of recovery and wellness (64). This is compounded for people from racialised communities, where racism, including exclusion from knowledge production, racial profiling, stereotyping, and ignoring linguistic and cultural diversity have contributed to the poor experience of mental health services by some communities, as reflected in the wealth of evidence of worse experience and outcomes than the majority population (65).

Values are a crucial dimension of culture as they are connected to ideas people hold about what is just and unjust, which is part of building trusting relationships. Cultural differences have been observed in the trust people have in public officials, with racialised groups reporting lower levels (66). This is noteworthy as trust is a component to help facilitate engagement in health provision. Similarly, racialised groups have different ideas about justice (67–69) and they are less likely to have trust and confidence in healthcare (70, 71), and they are more likely to perceive discrimination to be the reason for negative experiences (72). An understanding of these dimensions of identity and cultural differences are vital to the purpose of advocacy in recognition and redistribution of power and its overarching remit to protect and promote rights.

Fear is also a part of cultural beliefs which is relevant to mental health more generally and advocacy in particular. For Black groups especially, researchers have argued there is a fear of mental health services due to an expectation of being mistreated (73). It has been suggested that many Black people view psychiatric care as an extension of the way they are policed, and that mental health care is another strong arm of the state that enforces social control (74). When rights are felt to be so commonly abused, with an expectation of differential treatment, it is understandable that research shows how groups racialised as Black express a greater need for rights protection and enhancement (30).

Although there are observable cultural differences which run along blurred ethnic boundaries it remains vital to avoid reification and ascribe any sense of permanency to any racialised group. Culture is nevertheless significant to how we understand the differential experiences of racialised groups in mental healthcare. Ideas about rights, justice and service engagement are all part of cultural differences which are fundamental to how we construct the parameters of consideration for CAA.

### Race and racism

Concerns have also been expressed that a focus on cultural differences obfuscates from the need to address racism (75). Cultural competence has often been introduced to eliminate ethnic inequality and tackle racism. Here there is potentially a muddle in the use of terms as culture is conflated with race and ethnicity in the sense a culturally appropriate intervention can deal with ethnic inequalities and tackle racism. The inequalities that racialised groups experience are not only apparent because of cultural differences. Rather, racial bias, which is directly attached to observable physical differences, in particular skin colour, which provide stimuli which can be perceived negatively, contributing to differential treatment (66). Thus a focus on race and racism is essential to the very foundations of CAA.

There is little doubt that ideas related to race and culture overlap. Hatred can combine cultural and biological factors as they intertwined so evidently in the treatment of Jewish people prior to WWII (76, 77). In relation to mental health, the two concepts converge; barriers to help-seeking are not simply a cultural nuance, they are infused with a fear among racialised people about the care they will receive because of their racialised identity (17, 78). Cases of historical mistreatment of racialised people by the state more generally and by mental health services contribute to cultural beliefs and values about mental health services (79, 80). Nevertheless, race and racism need to be central to any conceptualisation of CAA. Race remains the basis for differential treatment (81). Healthcare professionals are not immune to making racial biased decisions (82). Racial bias is systemic, having a tendency to surface when operating in environments where quick decisions are required, under pressure in stressful conditions (66).

Given this context, challenging racism is crucial. It is possible to be aware of cultural differences, yet this does not necessarily translate into challenging services which are discriminatory. For this reason, emphasis on tackling all forms of racism needs to be incorporated within any model of CAA. Advocates need to be able to comprehend the effects of their own attitudes. They also need the ability to take action to prevent and address racism in all its guises: overt, covert and institutional. While culture may often be used as a byword to embody race in policy initiatives it is explicitly put forward here that CAA must have a role in addressing racism.

CAA that encompasses a direct challenge to racism is well situated in interpretations of advocacy as a liberatory practice, reflecting a concern with social justice and epistemic justice. People from racialised groups are particularly disadvantaged by their positioning at the intersection of state power and individual freedom, and between bio-psycho-social hegemony and alternatives narratives of distress. In this way, CAA should be focused on action that not only protects rights, in the form of abuses under the MH Act, but also on promoting rights, in the sense of broader empowerment and a challenge to institutional and social inequality. A broader range of outcomes that include social equality have been identified by African Caribbean men in relation to the purpose of advocacy (29). Hence attention to race, racism and power should be seen as fundamental components of CAA. This challenges a concept of IMHA restricted by statute to issue-focussed and transactional engagement with service users. It highlights the importance of individual advocacy being situated within collective action on structural inequalities.

### Culturally competent approaches

One of the earliest and most known definitions of cultural competence (CC), in relation to clinical practice, is that by Cross et al. (83). CC is analogous to culturally appropriate. This section draws on the former literature of CC, to provide a critique, and to elucidate components which are useful for framing culturally appropriate advocacy who refers to it as:

‘A set of congruent behaviours, attitudes, and policies that come together in a system, agency, or amongst professionals and enables that system, agency, or those professionals to work effectively in cross-cultural situations (p. 13)

Although Cross’ is the most widely cited there is no consensus on how CC should be defined. CC and other evolved forms of nomenclature (e.g., cultural awareness, cultural humility, cultural safety, culturally adapted, culturally responsive and culturally appropriate) each bring an element of conceptual confusion (34, 37). For example, Cross’ conceptualisation of CC has been critiqued as it makes no reference to the acquisition of information and knowledge about different cultures (84).

Taking a general overview there is recognition that CC is about producing better health outcomes through the acquisition of varied competencies at an individual level while integrating standards across policies and practices. These sentiments were broadly captured following a review of evaluated programs whereby CC was summarized as including:

“[A] set of skills or processes that enable mental health professionals to provide services that are culturally appropriate for the diverse populations that they serve”. (p.14) (85)

Most ways of understanding CC acknowledge the existence of, and the need to account for cultural differences. CC models indicate that for service users’ diverse needs to be met attention is required at three levels: structural, organizational and individual (86, 87). This goes beyond narrow understandings of CC which focus on workforce representation or ethnic matching. At the individual level CC typically includes the workforce having a particular set of skills and behaviors. For organizations, features include policies, practices and service design, while structural elements can include commissioning practices.

In most CC models the main aim is not to reflect the ethnic composition of a given population but to have a workforce that can operate to ensure equal outcomes by operating effectively in cross-cultural situations. To assist with developing a culturally competent organization initiatives at the individual level have generally comprised one of at least three components of learning: knowledge-based educational programs have focused on the provision of information such as diverging medical beliefs and practices or cultural interpretations of mental health and illness; attitude-based learning has sought to focus on issues of self-reflection and an exploration of bias, power and disparities; and skill based programs seek to improve communication skills and methods for interaction and how to elicit cultural differences in expectations.

However, CC which aim to build individual competencies have not come without criticism. Programs have often focused on either knowledge, skills or attitudes, missing other core elements of learning (88).

Approaches focused on attitudinal shifts, including cultural safety, cultural responsiveness or cultural humility (89) place emphasis on comprehending the historical, social, political and economic structures and power imbalances within which encounters between professional, and services users take place. While they entail self-reflection, encompassing ideas around humility, responsiveness and safety, they also hinge on the ability to listen and be respectful and open to patient’s stories and interpretations. However, concerns about how humility and sensitivity can be quantified, and the singular focus on only attitudinal-based learning have been raised (90). Much has also been made of competency as a construct. Models focused at an individual level have been critiqued for viewing competence as an end-ability whereas a more progressive stance is that of viewing it as an ongoing process (91, 92). Fernando critiques approaches which aim to describe professionals as being competent, as this would mean a person must (a) have sufficient knowledge about all cultures; (b) be fully aware of how to go about eliciting a person’s cultural background; and (c) possess attitudes of openness toward appreciating cultural differences (75). Hence, competency-based approaches are unlikely to be the standalone solution.

CC also requires organizational-level change. This can include positive action in recruitment where there is an identified need to ensure a more representative workforce alongside appropriate learning and coaching opportunities and robust attention to monitoring and evaluation. Cultural adaptations can also be considered at an organizational-level. Such adaptations to advocacy can help ensure existing practices respond to emerging needs (e.g., ethnic matching, change to a venue, modification of materials, changes to language metaphors or changes to types of practices including engagement approaches).

Individual and organizational level improvements should go hand-in-hand to improve the client’s experience. Beck and colleagues (93) who describe culturally responsive services in clinical care. They suggest how health professionals should be able to recognize and value diversity and draw on the support of team members to make adaptations to clinical care to be culturally affirming for the service user. Hinton and Patel (94) outline similar dimensions when referring to culturally sensitive approaches where the focus is on the overall context of the service user. What is of importance is that any approach requires focus at multiple levels, the outcome of which should be the increased ability of individuals and organizations to work effectively in cross-cultural situations.

### Defining culturally appropriate advocacy

With some exceptions, CC has been developed with clinical practice in mind (95). Even though this is the case, various components of CC are relevant to advocacy. Yet, as advocacy occupies a different space to other mental health professional roles there are several dimensions to consider before they can be transferred to the role of advocacy.

Indeed, a key differentiating factor of the advocate’s role is that it is not about providing care directly. Advocacy is about supporting and/or representing the service user voice and it is a role embedded in liberatory practice (20), with the aim of building service user’s capacity to self-advocate. Viewing advocacy within a framework of epistemic injustice means that attention has to be paid to the power relations in knowledge provision within which individual experience is located.

Independence from mental health services is another differentiating factor for advocacy, as it provides the conditions for scrutiny and for different understandings to the dominant discourse to emerge and to be promoted. Consequently, advocates can be viewed by statutory services as challenging and unduly critical (96). Also, as we will argue, CAA should not be limited to the narrow confines of ensuring the person has a voice (i.e., testimonial justice), as this risks ignoring the wider systemic injustices that have downgraded experience and the meaning of oppressive practices undermining good mental health.

These differences have implications for the skill sets associated with being culturally appropriate. Greater emphasis is placed on understanding and challenging wider institutional and structural inequalities. Representing and promoting service users’ voices at the junction between state power and individual freedoms requires specific knowledge around rights-based frameworks, capacity for self-reflection, and insight and willingness to be able to hold services to account for performance at the individual and system level. In acknowledging these differences, and as an attempt to identify its parameters, we put forward the following working definition:

Culturally appropriate advocacy entails an ongoing commitment by advocates and advocacy organisations, to respectively have the right knowledge, skills and sensitivities, and policies and practices, to challenge the abuse of rights and to work effectively in cross-cultural situations to protect and promote rights in order to achieve greater equality, and ultimately social justice.

This definition builds on ideas within CC and encompasses the need to pay attention to rights promotion and protection. It recognizes the need to focus on individual and institutional factors and how bias operates iteratively at different levels (97). While the definition draws on the need for varied competencies, it overcomes some of the conceptual challenges already identified in relation to definitions of CC. By focusing on the term appropriate, rather than competent, it elicits the expectations placed on the advocate to provide culturally affirming support but to also recognize the journey will never be complete. In this way, the definition overcomes the perception associated with competency which presupposed that a static end-goal can be reached by placing emphasis on the need to be open to ongoing learning.

### Defining elements of culturally appropriate advocacy

This section will operationalize some of the key conceptual elements pertaining to the role of CAA. Considerations are defined at three levels for an individual advocate, advocacy organizations and structural factors.

### Conceptual framing learning domains for culturally appropriate advocates

Based on a review of advocacy provision by the authors (5) and an evaluation of a culturally appropriate training pilot (98), a learning framework for culturally appropriate advocacy is an area in need of development. Similar frameworks (99) have been developed and applied to other professions within mental healthcare yet few of these have been evaluated, and advocacy has not been an area of consideration.

For advocates, we put forward the suggestion for learning across three domains: knowledge, sensitivities (attitude) and skills; the validity and interactive nature of which have some basis (91). While some models only focus on learning across one domain (e.g., knowledge), a more comprehensive and holistic approach is put forward to encourage wider learning and application. In accordance with the strength of evidence of effectiveness, the approach to learning should incorporate theory and research (100), and center the lived experience perspective. The aim of this section is not to document intricate parts of each learning domain. Rather it is to outline an approach which considers knowledge, sensitivities and skills as part of a culturally appropriate advocate’s role.

We use knowledge to infer the cognitive element of any culturally appropriate approach that focuses on the acquisition of information. This includes, for example, advocates developing an understanding of broad aspects of culture and its relevance to (mental) health (e.g., pluralistic help-seeking); the social determinants of poor mental health; the ways in which bias manifests itself and mechanisms used to mitigate it. It is important to re-emphasize, this is not about having knowledge about all cultures, but developing an information base continuously about the specific ethnic groups in the geographic location where advocates operate. Further areas of knowledge include the need to develop an understanding of historical (ie. slavery and colonialism) and present power dynamics including personal power and culture (100). Research argues for an informed and deeper understanding of race, culture and ethnicity as socially constructed entities (5, 99) and the importance of having an intersectional lens and a non-essentialist approach. Other areas of knowledge acquisition may include the social and psychological effects of racism, community-based approaches and alternatives to mainstream support, and the contribution of social context (in hospital and in the community) to mental distress (89, 101), rights-based training and the manifestation of epistemic injustice in mental healthcare. Indeed, the areas covered are not intended to be exhaustive, but to illuminate knowledge as a learning domain and some of its constituent parts.

Sensitivity points toward the affective aspects of an advocate’s role, and another domain of focus. While people can acquire knowledge, the right mindset needs to be in place for an advocate to use their learning and to be respectful of racialised differences. Hence, sensitivity is about the desire and effort of advocates which involves an attitude toward appreciating diversity. While many models use the construct attitude, we use sensitivity to encompass attitudes, humility, perceptions, values and aspects of behaviors. CC models have been critiqued for not placing enough emphasis on power and have promoted ideas about cultural safety and cultural humility (102). When using the term sensitivity we promote the necessity for self-reflection, to include an analysis of personal and structural power. This encapsulates ideas within cultural safety and humility models for there to be strength of awareness relating to socio-cultural factors, to facilitate a situation whereby advocates see one-self and their organizations as a cultural entity and that of the populations served. It is acknowledged here that sensitivity is a difficult metric to measure and therefore independent monitoring efforts are necessary at the organizational level, from client input, to reviewing advocacy performance.

The skills domain of the framework focuses attention on the way knowledge and sensitivities can be enacted. This can include the skill to identify when a person may need an interpreter and acting on this, or the skill and capacity to build positive and trusting relationships. It can include the communication skills to be able to elicit whether a person’s cultural background has been factored into decision-making. Advocacy involves the skills to not only detect racism but to be proactive, involving critical thinking, including the commitment to critique hegemonic models of mental distress (103), as an element of an advocate’s composite skills.

It is possible to explicate how the three learning domains (i.e., knowledge, sensitivity and skills) function interactively. If we take the scenario of a Somali man who feels he has experienced spiritual possession. An advocate needs to access knowledge to understand the significance of different cultural groups and their diverse explanatory models of mental health and self-defined outcomes. They would need to be sensitively attuned to view this as important. Advocates would also need the necessary skill to support and/or represent their client and engage with mental health professionals to encourage them to incorporate their explanatory framework into assessment and care, aiming to build a more developed understanding of client’s valued outcomes and how these can be achieved. This example illustrates how the three learning domains can be applied to the role of an advocate. It is also starts the process of conceptually framing the role of CAA at an individual level, and how this can be aligned with the CAA definition provided.

### Conceptual framing culturally appropriate advocacy organizationally

While advocates can play a vital role in resolving issues of epistemic injustice, attention also needs to be focused at the level of the advocacy organization. Any conceptual framing of CAA must expand beyond the level of the individual to include various domains at the organizational level, be this different advocacy delivery models, appraising outputs and outcomes, alongside policies and procedures.

The landscape of mental health advocacy provision is dominated by providers that specialize in a range of advocacy and their capacity and relationships with local communities is often under-developed. The following is particularly oriented toward improving the cultural appropriateness of their provision but should not be interpreted as precluding provision by culturally specific organizations which are likely to have a strong foundation in action to achieve racial justice.

Any conceptual framing of CAA must acknowledge the need for functioning organizational policies and procedures. It is by no means out of the realms of possibility for organizations to believe they are implementing progressive policies and procedures when their services can be discriminatory, for example, by inadvertently restricting service access. Embedded systems of data collection are not sufficient without appropriate evaluation, which should in turn inform service design. Service design and outcomes, based on models involving co-production with different groups and based on cultural differences among the service user population, should be common practice wherever appropriate. Other organizational factors, such as working environments, which may not be conducive for racialised people, need to be part of thinking in culturally appropriate ways. This may entail considering the racialised trauma experienced by advocates against the background of issues they encounter in their work.

CC includes an emphasis on organizational values and governance, considering the extent to which equality more generally or race equality in particular, are apparent in documentation, leadership and investment. Other domains include: communication, the need for interpretation or translation services in both written and oral forms to successfully engage and provide support; staff development, involving training, support and supervision, and whether positive action principles, especially around succession planning and client engagement, are implemented. CC frameworks also incorporate a domain centered on organizational infrastructure. This relates to workforce diversity, technology, it could also include linkages and alliances with experts in the field of CC, and partnership with Black led organizations. These are elements which can be transferred to any conceptual framing of CAA.

Service design is linked to the organizational conception of CAA. This can consider facility characteristics, including the access, availability and acceptability of provision, and the environment and location. This is pertinent to models which are viewed as culturally or ethnically specific. Service design can include partnership work, such as, targeted provision for organizations which have a remit to support individuals from specific ethnic backgrounds. Such organizations will have an advantage in terms of hermeneutic justice, although achieving this may well be constrained by the wider social and organizational context. These organizations, typically, undertake collective advocacy although they have been increasingly marginalized in the provision of formal individual advocacy. However, group advocacy is one element of service design which may form a key part of CAA approaches, and thus a consideration in its conceptual framing. It also provides the means to challenge the conditions of hermeneutical injustice. Solidarity is critical to generating collective hermeneutic resources, and by offering a space for interpretative and shared meaning-resources and concepts to be understood, developed and expressed, group advocacy can assist people historically excluded and hermeneutically marginalized (104). Racialised service users can utilize group advocacy settings to frequently come together using their lived experience to develop and share tools and strategies for interpretation and action. It is therefore necessary for advocacy organizations to review the delivery approaches and explore different methods of engagement to amplify service user’s voice, and to use case information to inform other system players, including commissioners and NHS providers. Such actions and design considerations are fundamental to how CAA is conceived at an organizational level.

### Structural and systemic factors

While the focus has been on individual and organizational factors, advocacy organizations are subject to the caprices of processes operating structurally. Austerity and a shift to neo-liberal managerialism can all impinge and place restrictions on the way advocacy operates, and mental health practices in general.

The role of commissioners also falls at this macro level of operation. Commissioners determine the scope and nature of advocacy provision and their role should not be under-estimated. As noted elsewhere (105), it is vital that advocacy services are based on engagement with, and a developed understanding of, the diversity of the local population. The very structure of advocacy, for instance case-based work, can be informed by the priorities of commissioners. At the same time, commissioners need to be attuned to racism and how bias operates through commissioning processes that may systematically disadvantage smaller community organizations that have both relationships, knowledge and sensitivity to provide independent advocacy services for specific populations. In the shifting world of commissioning and a re-energized focus on addressing inequalities in mental health, there needs to be accountability and transparency in commissioning advocacy.

A structurally specific framing of CAA acknowledges macro level factors, including the environment, the willingness of mental health trusts to engage and financial constraints within which advocacy organizations find themselves. Reduced finances can stymie approaches to pro-active engagement with service users as it impacts resource capacity. Similarly, opportunities for meaningful monitoring and evaluation at an organizational level can be hindered by the financial envelopes allocated by commissioners, and what is prioritized by local authorities. These matters are brought to the fore in the present framing of CAA as they impact directly on provision and have implications for epistemic injustice.

## Discussion

Addressing epistemic injustice in mental healthcare is a clear priority. This is particularly the case for racialised people. Culturally appropriate advocacy has a key role in addressing this imbalance within power relations. We argue, that any framing of CAA needs to acknowledge the importance of culture against a background of western hegemony, while equally recognizing racism in all its guises.

The conceptual framing of CAA offered here is one the authors hope will promote further discussion. It offers a scaffolding of consideration at individual, organizational and structural levels, through which CAA can be situated. It does so through the lens of challenging epistemic injustice. Advocacy has already demonstrated it plays a role in relation to testimonial injustice. The focus on knowledge, skills and sensitivities across areas of racial, ethnic and cultural difference, conceptualized at an individual level consolidates advocacy’s emphasis on testimonial injustice, while paying attention to the egregious position of racialised people in mental health care.

CAA has the potential to plug an evidenced gap relating to hermeneutic injustice. To do so, minimum efforts are required at all the levels at which CAA is framed. A willingness from advocates to provide a space for the development of shared meaning and resources must go together with a shift in how advocacy organisations operate, and the means with which they can operate.

When framing CAA at an organizational level, there is likely to be a strong call for practical considerations about how lawful requirements under the Equality Act 2010 can be met. This entails a real need to review service uptake, paying attention to feedback and appraising models of engagement, and where necessary adopting pro-active models of engagement. However, taking steps toward hermeneutic justice will require greater steps toward collectivism and solidarity. Part of this may come from enabling a collective voice, through approaches such as group advocacy, increased investment in and support for Black-led approaches; thus stretching the narrowly confined conceptions of IMHA.

Attempts to redress the resource deficit through solidarity and collectivism, must be supported through commissioning, and must have a channel to inform and drive system-wide change. For CAA to be effective there needs to be a listening ear on the part of Mental Health Trusts. This is a structural consideration in the framing of CAA. It is about leaders within Mental Health Trusts being receptive and accountable. There is some reason to be positive that changes in the Mental Health Act can provide the necessary apparatus. The Patient and Carer’s Race Equality Framework (PCREF), another article in this special edition, may on the one hand provide the means to appraise delivery to racialised populations at a system-wide level. At the same time, it can provide the conduit through which CAA can inform the system about the experiences of people in mental health services, contributing to institutional accountability. However, codesign and partnerships with community and voluntary organizations for people from racialised communities, in full recognition of their key role in prevention and early intervention, will go some way to improving experience and outcomes and addressing wider social determinants.

## Conclusion

CAA was a recommendation of the Independent Review of the Mental Health Act. It is unclear how the term culturally appropriate is conceptualized in relation to advocacy. Existing cultural competency frameworks do not encompass the specific functions of advocates and advocacy organizations and their position as a liberatory practice. This paper argues that advocacy and culturally appropriate practices are especially relevant to racialised people. It clarifies the importance of culture, race and racism to the role of advocacy, and draws on cultural competency models from across clinical practice to develop a conceptual framing of cultural appropriate advocacy.

CAA has implications for people experiencing mental health problems and racism, and professionals involved in care and treatment. If the potential of culturally appropriate advocacy to improve the experience of people from racialised communities in respect of mental health services in general and mental health legislation in particular, then we need to be clear that part of its role will be to address issues relating to race and racism. It is self-evident that while protecting and safeguarding rights in this context is essential, promoting the substantive rights of people from racialised populations will go further in achieving their better mental health and recovery and make major strides toward equality, greater social and epistemic justice.

## Author contributions

All authors listed have made a substantial, direct, and intellectual contribution to the work and approved it for publication.

## Conflict of interest

The authors declare that the research was conducted in the absence of any commercial or financial relationships that could be construed as a potential conflict of interest.

## Publisher’s note

All claims expressed in this article are solely those of the authors and do not necessarily represent those of their affiliated organizations, or those of the publisher, the editors and the reviewers. Any product that may be evaluated in this article, or claim that may be made by its manufacturer, is not guaranteed or endorsed by the publisher.

## Footnotes

^1^We use the term racialised to acknowledge the historical, cultural, political and economic processes resulting in the construction and reproduction of groups based on racial identity. This is used in preference to the term Black, Asian and Minority Ethnic.
